# Skeletal Muscle Depletion Predicts the Prognosis of Patients With Hepatocellular Carcinoma Treated With Radiotherapy

**DOI:** 10.3389/fonc.2019.01075

**Published:** 2019-10-15

**Authors:** Joongyo Lee, Yeona Cho, Sangjoon Park, Jun Won Kim, Ik Jae Lee

**Affiliations:** Department of Radiation Oncology, Yonsei University College of Medicine, Seoul, South Korea

**Keywords:** hepatocellular carcinoma, sarcopenia, radiotherapy, treatment outcome, survival

## Abstract

**Background:** Sarcopenia is gaining attention as a poor prognostic factor for various types of malignancies. This study evaluated the prevalence and prognostic significance of sarcopenia and its association with survival in hepatocellular carcinoma (HCC) patients who underwent radiotherapy (RT) to the primary site.

**Materials and Methods:** Between January 2009 and November 2016, 156 patients with HCC that underwent RT to the liver were retrospectively studied. Sarcopenia was defined as an L3 skeletal muscle index of <49 cm^2^/m^2^ for men and <41 cm^2^/m^2^ for women as proposed by Korean-specific cut-off value. Sarcopenia was identified pre- and post-RT (within 3 months from the end of RT).

**Results:** Pre-RT sarcopenia occurred in 99 (63.5%) patients and was significantly associated with higher levels of protein induced by vitamin K absence or antagonist-II (PIVKA-II), lower percentage of overweight/obesity (body-mass index), higher percentage of previous systemic chemotherapy, and lower total RT dose. At a median follow-up of 9.3 months, median overall survival (OS) was significantly lower in patients with pre-RT sarcopenia than in those without (7.1 vs. 15.3 months, *p* < 0.001). In multivariate analysis [reporting hazard ratio (HR): 95% confidence interval (CI)], albumin-bilirubin score (2.35: 1.33–4.17; *p* = 0.003), total dose (0.44: 0.27–0.71; *p* = 0.001), and pre-RT sarcopenia (2.38: 1.53–3.70; *p* < 0.001) were independent OS prognostic factors. Among patients without pre-RT sarcopenia, 20 newly developed sarcopenia after RT and showed significantly lower OS compared to those without sarcopenia after RT (*n* = 35) (median 14.1 vs. 17.5 months, *p* = 0.018). Multivariate logistic regression analysis [reporting odds ratio (OR)] demonstrated older age (310.190; *p* = 0.007), Child-Pugh classification B or C (15.239; *p* = 0.047), higher alpha-fetoprotein (128.486; *p* = 0.008), higher PIVKA-II (118.536; *p* = 0.027), and larger planning target volume (51.310; *p* = 0.026) as significant factors for newly developed post-RT sarcopenia.

**Conclusion:** Newly developed sarcopenia after RT, as well as pre-RT sarcopenia, were associated with poor survival for HCC patients who underwent RT to the liver. This result suggests the possibility that early intervention such as nutritional support and exercise therapies before and during RT could prevent muscle wasting and may be effective in improving the prognosis of HCC patients.

## Introduction

Hepatocellular carcinoma (HCC) is one of the most common malignancies with poor prognosis, and the incidence of HCC has been steadily rising (1). With the progress in the diagnosis and treatment of HCC, local treatment such as resection, transcatheter arterial chemoembolization (TACE), transcatheter arterial chemotherapy infusion (TACI), and radiofrequency ablation (RFA) became the standard treatment, resulting in great improvement in survival and disease control. In addition, conventional radiotherapy (RT) or stereotactic body RT (SBRT) achieved substantial tumor regression and survival as reported in various studies (2–5). Despite the development of various treatment strategies, the prognosis of HCC is still poor, and several studies are underway to find the prognostic factors to improve outcome.

Poor general condition, low hepatic functional reserve, and protein energy malnutrition causing impaired immunity and dysregulated metabolism lead to poor long-term prognosis of HCC (6, 7). The concept of cachexia has been commonly used to describe the general condition of such cancer patients. The agreed diagnostic criterion for cachexia was weight loss >5%, or weight loss >2% in individuals already showing depletion according to current bodyweight and height [body-mass index (BMI) <20 kg/m^2^] or skeletal muscle mass (8). However, cachexia can sometimes be misdiagnosed because the assessment of weight change depends on patient's response during a physical examination (9). Furthermore, changes in body weight do not fully reflect body composition change, and weight loss is uncertain for patients with large tumor, pleural effusion, or severe body edema (10). Therefore, a more qualified indicator of this wasting condition is needed to predict the prognosis of HCC.

Sarcopenia is defined by the loss of skeletal muscle mass, quality, and strength. The causes of sarcopenia include aging, disuse (poor performance status), nutritional deficiencies, advanced organ failure, inflammatory disease, and cancer treatment-related toxicities (11). Noteworthy is that there is evidence that sarcopenia is prevalent in cancer patients, regardless of stage of disease and nutritional status (12) and it is identified as a poor prognostic factor for various types of malignancies (13–16). Particularly, sarcopenia occurs frequently in cases of HCC after treatment (17–19) and many studies on the association of HCC prognosis with sarcopenia were performed. These studies have shown that sarcopenia was an independent predictor of mortality in patients with HCC following partial hepatectomy, intra-arterial HCC therapy, or after sorafenib (17–20). However, few studies have investigated that sarcopenia is an independent predictor of prognosis in patients receiving RT for HCC.

In this study, we investigated the prevalence and prognostic significance of sarcopenia in patients that underwent RT for HCC. In addition, we also evaluated the associated factors that induce sarcopenia after RT.

## Materials and Methods

### Patient Selection

The medical records and radiology database at Gangnam Severance hospital were searched for patients with primary HCC stage I-IVB (Liver Cancer Study Group of Japan [LCSGJ]) that underwent RT to the liver from January 2009 to November 2016. HCC was diagnosed based on typical dynamic study findings of enhanced staining in the early phase and attenuation in the delayed phase (21). Patients who did not receive RT directly to the liver or those who did not complete RT were excluded. We evaluated 156 patients who were eligible for our study.

### Treatment

In this study, the aim of RT was classified as definitive, salvage, and palliative. Definitive aim meant treating the first diagnosed HCC for the purpose of complete remission. Salvage aim meant retreatment if previous local treatment of HCC failed. Palliative aim was defined as treatment for symptom control when cure was difficult to achieve.

Patients were treated with RT alone or concurrent chemoradiotherapy (CCRT). In the CCRT group, concurrent hepatic arterial infusion of 5-fluorouracil (5-FU) was delivered during RT. Most patients received conventional RT (30–70 Gy in 10–30 fractions) with 3-dimensional conformal RT (3D-CRT) or intensity-modulated RT (IMRT), and a small number of patients received SBRT (36–60 Gy in four fractions).

Treatment before RT was classified as surgery, systemic chemotherapy, or local treatment (TACE or TACI or RFA). All surgeries were partial hepatectomies, and most of the patients undergoing systemic chemotherapy received sorafenib regimen.

### Definition of Sarcopenia

Sarcopenia was identified at two-time points: pre- and post-RT. Pre-RT sarcopenia was diagnosed with data from the simulation computed tomography (CT) prior to RT, while post-RT sarcopenia was diagnosed with data from the follow-up CT taken within 3 months from the end of RT.

**Figure 1:**
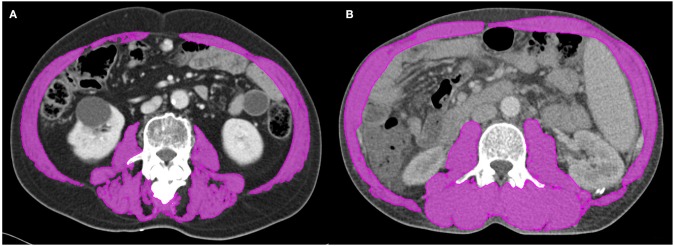
Segmented computed tomography (CT) images of patients with **(A)** and without **(B)** sarcopenia who had similar body mass indices. Skeletal muscle was measured using the MIM Vista software (MIM corp., version 6.1, OH, USA).

We retrospectively measured the cross-sectional area of muscle at the level of the third lumbar vertebra using baseline CT images. The muscles were quantified within a Hounsfield unit (HU) range of −29 to +150, and muscle boundaries were manually corrected as needed (Figure 1) (22, 23). Then, we calculated the L3 skeletal muscle index (L3-SMI) as follows:

$$\begin{array}{l} \frac{\text{The cross}-\text{sectional area of muscle at L}3\text{ spine level on CT }\left(\text{c}{\text{m}}^{2}\right)}{\text{Height }\times \text{ Height }\left({\text{m}}^{2}\right)} \end{array}$$

Sarcopenia defined by international consensus of cancer cachexia was <55 cm^2^/m^2^ for men and <39 cm^2^/m^2^ for women (8). However, this is based on studies among Westerners, and different standards are needed for Asians with differences in their intrinsic muscles. Therefore, we used the Korean-specific cut off values defined in other studies. Sarcopenia was defined as an L3-SMI of <49 cm^2^/m^2^ for men and <41 cm^2^/m^2^ for women as proposed by Korean-specific cut off values (24).

### Data Collection

Demographic and treatment characteristics of individual patients were obtained from the medical records, including age at diagnosis, sex, tumor stage, liver enzymes, Child-Pugh classification, tumor marker, and treatment before RT. All patients underwent initial and response evaluation based on computed tomography (CT) or magnetic resonance imaging (MRI).

Height and weight were recorded at first visit to our department. The BMI grouping based on the World Health Organization criteria is as follows: BMI <18.5 kg/m^2^ (underweight), BMI 18.5–25 kg/m^2^ (normal weight), BMI 25–30 kg/m^2^ (overweight), and BMI ≥ 30 kg/m^2^ (obesity).

Information was collected on the following variables to analyze the prognostic factors for overall survival (OS): age; sex; viral infection (hepatitis B virus, hepatitis C virus); total dose of RT; RT modality; planning target volume (PTV); CCRT; Child-Pugh classification (A vs. B + C); HCC stage at the time of RT; portal vein tumor thrombus; prior treatment (resection, chemotherapy, TACE or TACI or RFA); alpha-fetoprotein (AFP, cut off value 20 ng/mL); protein induced by vitamin K absence or antagonist-II (PIVKA-II, cut off value 40 mAU/mL); serum total protein (cut off value 6.0 g/dL); serum albumin level (cut off value 3.4 g/dL); albumin-bilirubin (ALBI) grade; and BMI. The ALBI score was derived from the formula (log_10_ bilirubin [μmol/L] × 0.66) + (albumin [g/L] × −0.0852).

### Statistical Analysis

Baseline characteristics of patients with and without sarcopenia were compared via the Pearson χ^2^ test for categorical data and independent *t*-test for continuous data.

OS was defined as the time from start of RT until death or last patient contact. OS was analyzed using the Kaplan-Meier method and log-rank test. Univariate and multivariate analyses for OS were performed with the Cox proportional hazards model. Multivariate survival analysis was conducted using the Cox proportional hazards regression, and variables with a *p* < 0.10 in univariate analysis were included. The multivariate analysis for risk factor of post-RT sarcopenia was also performed using logistic regression analysis.

All *p*-values lower than 0.05 were considered statistically significant. Statistical analysis was performed using IBM SPSS, version 23.0 (IBM Corp., Armonk, NY, USA).

## Results

### Patient and Treatment Characteristics

The baseline characteristics of total 156 patients are listed in Table 1. For all the patients, the median L3-SMI for pre-RT was 46.4 cm^2^/m^2^ (range 29.5–66.6 cm^2^/m^2^) in men and 38.4 cm^2^/m^2^ (range 26.1–57.5 cm^2^/m^2^) in women. According to Korean-specific cut off standard, 99 patients (63.5%) had pre-RT sarcopenia; 81 patients (81.8%) were men and 18 (18.2%) women.

**Table 1:** Characteristics of total patients with HCC and patients with HCC according to the presence of pre-RT sarcopenia.

		**Total**		**Pre-RT sarcopenia**				
				**Non-sarcopenic**		**Sarcopenic**		***p*-value**
		***N* = 156**	**%**	***N* = 57**	**%**	***N* = 99**	**%**	
Age (years)	Median	59		57		61		0.592
	Range	23–87		34–80		23–87		
Sex	Male	128	82.1	47	82.5	81	81.8	0.920
	Female	28	17.9	10	17.5	18	18.2	
Etiology	HBV	113	72.4	39	68.4	74	74.7	0.571
	HCV	14	9.0	7	12.3	7	7.1	
	Non-B, non-C	29	18.6	11	19.3	18	18.2	
HCC stage	I	3	1.9	2	3.5	1	1.0	0.372
	II	15	9.6	6	10.5	9	9.1	
	III	48	30.8	18	31.6	30	30.3	
	IVA	69	44.2	24	42.1	45	45.5	
	IVB	21	13.5	7	12.3	14	14.1	
Portal vein tumor thrombus	Yes	93	59.6	35	61.4	58	58.6	0.730
	No	63	40.4	22	38.6	41	41.4	
Child-Pugh score	A	96	61.5	40	70.1	56	56.6	0.115
	B	57	36.5	16	28.1	41	41.4	
	C	3	1.9	1	1.8	2	2.0	
AFP (ng/mL)	Median	210.4		50.2		296.9		0.279
	Range	1.3–54,000		1.8–54,000		1.3–54,000		
PIVKA-II (mAU/mL)	Median	1108.0		446.0		2000.0		0.001
	Range	4.5–185,072		4.5–51,851		9.0–185,072		
Total protein (g/dL)	Median	6.8		7.0		6.7		0.027
	Range	4.8–9.3		5.4–8.5		4.8–9.3		
Albumin (g/dL)	Median	3.5		3.6		3.4		0.015
	Range	2.4–4.9		2.5–4.8		2.4–4.9		
ALBI score	Median	−2.18		−2.25		−2.14		0.081
	Range	−3.55 to −0.64		−3.37 to −0.91		−3.55 to −0.64		
BMI (kg/m^2^)	Underweight	4	2.6	0	0	4	4.0	0.000
	Normal weight	104	66.7	26	45.6	78	78.8	
	Overweight	41	26.3	25	43.9	16	16.2	
	Obesity	7	4.5	6	10.5	1	1.0	
Previous treatment	Surgery	15	9.6	7	12.3	8	8.1	0.392
	Chemotherapy	15	9.6	1	1.8	14	14.1	0.011
	TACE/TACI/RFA	94	60.3	37	64.9	57	57.6	0.367
	None	55	35.3	18	31.6	37	37.4	0.466
RT aim	Curative	113	72.4	45	78.9	68	68.7	0.167
	Palliative	43	27.6	12	21.1	31	31.3	
Treatment scheme	RT alone	75	48.1	31	54.4	44	44.4	0.231
	CCRT	81	51.9	26	45.6	55	55.6	
RT modality	3D-CRT	52	33.3	21	36.8	31	31.3	0.481
	IMRT	104	66.7	36	63.2	68	68.7	
RT dose	Median	52.1		52.9		52.1		0.029
(EQD2, α/β = 10)	Range	30.0–125.0		40.0–125.0		30.0–99.2		
PTV (cc)	Median	735.4		627.21		829.37		0.017
	Range	15.0–5851.2		15.0–3347.1		30.3–5,851.2		
RT scheme	Conventional RT	153	98.1	55	96.5	98	99.0	0.554
	SBRT	3	1.9	2	3.5	1	1.0	

*HCC, Hepatocellular carcinoma; RT, Radiotherapy; HBV, Hepatitis B virus; HCV, Hepatitis C virus; AFP, Alpha-fetoprotein; PIVKA-II, Proteins induced by vitamin K absence or antagonist-II; ALBI, Albumin-bilirubin; BMI, Body mass index; TACE, Transcatheter arterial chemoembolization; TACI, Transcatheter arterial chemotherapy infusion; RFA, Radiofrequency ablation; CCRT, Concurrent chemoradiation therapy; 3D-CRT, 3-Dimensional conformal radiation therapy; IMRT, Intensity-modulated radiation therapy; EQD2, Equivalent dose in 2 Gy fractions; PTV, Planning target volume; SBRT, Stereotactic body radiotherapy*.

Of the 156 patients, 90 patients had stage IV disease, of whom 21 patients had distant metastasis. Most patients were Child-Pugh class A (*n* = 96, 61.5%) and few were Child-Pugh class C (*n* = 3, 1.9%). According to the BMI, patients with normal weight were the most frequent (*n* = 104, 66.7%), followed by those who were overweight (*n* = 41, 26.3%). Pre-RT sarcopenia group had higher PIVKA-II (*p* = 0.001), lower serum total protein level (*p* = 0.027), lower serum albumin level (*p* = 0.015), and lower percentage of overweight/obesity BMI (*p* < 0.001). Although not statistically significant, pre-RT sarcopenic patients had poorer Child-Pugh class and more advanced stage.

Regarding treatment characteristics, 81 patients (51.9%) were treated with concurrent intra-arterial chemotherapy, and 75 patients (48.1%) were treated with RT alone. Most of the patients (*n* = 153, 98.1%) received conventional RT, and only three patients (1.9%) received SBRT. Median prescribed equivalent dose in 2 Gy fractions, assuming α/β = 10 (EQD210) was 52.1 Gy. Compared with the group without pre-RT sarcopenia, the pre-RT sarcopenia group had a higher percentage of patients with previous systemic chemotherapy (*p* = 0.011), lower total prescribed dose (*p* = 0.029), and larger PTV (*p* = 0.017). Pre-RT sarcopenic patients had more RT for palliative treatment than pre-RT non-sarcopenic patients, even though no statistically significant difference was found.

### Analysis of Survival

**Figure 2:**
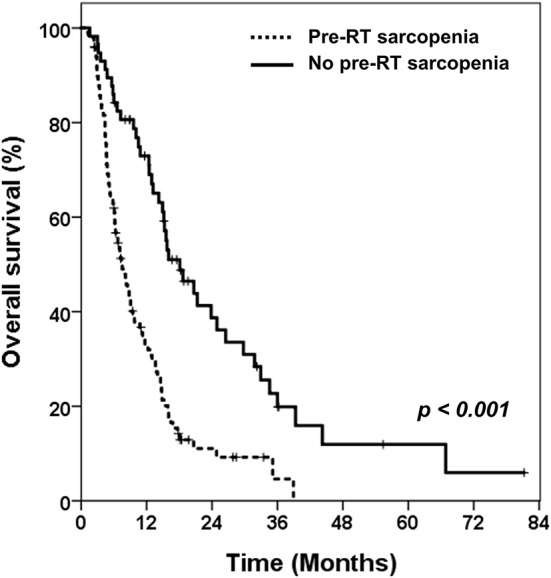
Kaplan-Meier curves of overall survival in patients with or without pre-RT sarcopenia.

Over a median follow-up duration of 9.3 months (range 1.2–81.2 months), 125 patients died. Patients in the pre-RT sarcopenia group showed poorer OS than those in the pre-RT non-sarcopenia group (median OS: 7.1 months vs. 15.3 months, *p* < 0.001; Figure 2).

**Figure 3:**
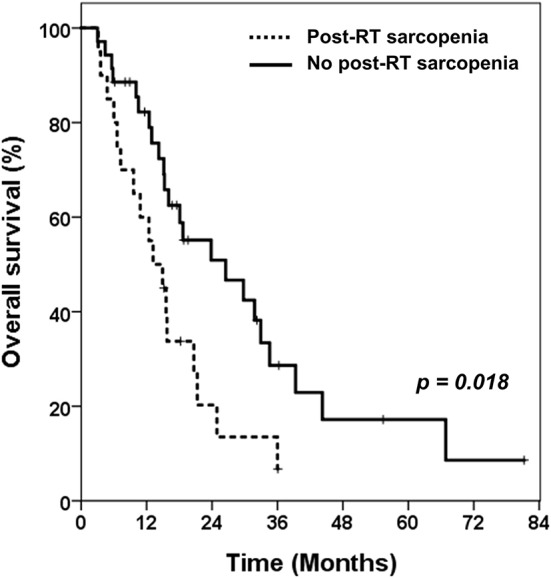
Kaplan-Meier curves of overall survival in patients without pre-RT sarcopenia. Comparing overall survival between patients with newly developed sarcopenia after receiving RT and patients who still had no sarcopenia after RT. RT, radiotherapy.

The evaluation of post-RT sarcopenia was available in 148 patients. Only four patients in pre-RT sarcopenia group had overcome the sarcopenia after RT. Among the 57 patients who did not have sarcopenia before RT, 20 developed sarcopenia after RT (35.1%). Compared to those without sarcopenia after RT (*n* = 35), these 20 patients showed significantly lower OS (median 14.1 vs. 17.5 months, *p* = 0.018, Figure 3). The prognosis of these patients was as poor as those with sarcopenia before RT.

### Prognostic Factors for Survival

The results of Cox proportional hazard regression analysis for OS are shown in Table 2. In univariate analysis, advanced stage at the time of RT (HR: 2.63; 95% CI: 1.28–5.40; *p* = 0.009); Child-Pugh classification B or C (HR: 1.46; 95% CI: 1.02–2.10; *p* = 0.041); higher AFP (HR: 1.63; 95% CI: 1.10–2.41; *p* = 0.015); lower serum albumin level (HR: 0.51; 95% CI: 0.35–0.73; *p* < 0.001); higher ALBI score (HR: 2.01; 95% CI: 1.47–2.75; *p* < 0.001); lower total dose of RT (HR: 0.45; 95% CI: 0.31–0.64; *p* < 0.001); and larger PTV (HR: 1.02; 95% CI: 1.00–1.04; *p* = 0.021) were identified as poor prognostic factors for OS. Pre-RT sarcopenia (HR: 2.62; 95% CI: 1.76–3.89; *p* < 0.001) was also significant for OS.

**Table 2:** Univariate and multivariate analysis for OS.

	**Overall survival**			
	**Univariate analysis**		**Multivariate analysis**	
	**HR (95% CI)**	***p*-value**	**HR (95% CI)**	***p*-value**
Age (<65 vs. ≥65 years)	0.98 (0.66–1.46)	0.92		
Sex (Male vs. Female)	0.81 (0.50–1.31)	0.383		
Viral infection (No vs. Yes)	1.28 (0.81–2.03)	0.30		
HCC stage (I + II vs. III + IV)	2.63 (1.28–5.40)	0.00	1.75 (0.79–3.85)	0.166
Portal vein tumor thrombus (No vs. Yes)	1.19 (0.83–1.72)	0.34		
Child-Pugh class (A vs. B + C)	1.46 (1.02–2.10)	0.04	0.95 (0.60–1.52)	0.842
AFP (<20 ng/mL vs. ≥20 ng/mL)	1.63 (1.10–2.41)	0.01	1.03 (0.65–1.64)	0.888
PIVKA-II (<40 mAU/mL vs. ≥40 mAU/mL)	1.37 (0.87–2.14)	0.17		
Total protein (<6.0 g/dL vs. ≥6.0 g/dL)	0.73 (0.43–1.26)	0.264		
Albumin (<3.4 g/dL vs. ≥3.4 g/dL)	0.51 (0.35–0.73)	0.000	0.98 (0.55–1.75)	0.937
ALBI score	2.01 (1.47–2.75)	0.000	2.35 (1.33–4.17)	0.003
BMI (<25 vs. ≥25 kg/m^2^)	1.11 (0.78–1.59)	0.548		
Prior treatment				
Surgery	0.58 (0.31–1.08)	0.084	0.87 (0.44–1.74)	0.698
Chemotherapy	1.63 (0.91–2.93)	0.099	0.87 (0.44–1.74)	0.900
TACE or TACI or RFA	1.13 (0.78–1.63)	0.512		
Treatment scheme (RT alone vs. CCRT)	1.09 (0.77–1.55)	0.637		
RT modality (3D CRT vs. IMRT + Tomo)	0.73 (0.51–1.06)	0.097	0.96 (0.58–1.58)	0.869
Total dose (EQD2 <52.1 Gy vs. ≥52.1 Gy)	0.45 (0.31–0.64)	0.000	0.44 (0.27–0.71)	0.001
PTV	1.02 (1.00–1.04)	0.021	1.01 (0.99–1.03)	0.399
Pre-RT sarcopenia (no vs. yes)	2.62 (1.76–3.89)	0.000	2.38 (1.53–3.70)	0.000
Newly developed sarcopenia after RT (No vs. Yes)^*^	2.16 (1.12–4.16)	0.021	2.53 (1.28–5.02)	0.008

* *Univariate and multivariate analysis for overall survival in patients without pre-RT sarcopenia*.

*OS, overall survival; HR, Hazard ratio; CI, Confidence interval; HCC, Hepatocellular carcinoma; AFP, Alpha-fetoprotein; PIVKA-II, Proteins induced by vitamin K absence or antagonist-II; ALBI, Albumin-bilirubin; BMI, Body mass index; TACE, Transcatheter arterial chemoembolization; TACI, Transcatheter arterial chemotherapy infusion; RFA, Radiofrequency ablation; RT, Radiotherapy; CCRT, Concurrent chemoradiation therapy; 3D-CRT, 3-Dimensional conformal radiation therapy; IMRT, Intensity-modulated radiation therapy; EQD2, Equivalent dose in 2 Gy fractions; PTV, Planning target volume*.

Then we performed multivariate analysis; ALBI score (HR: 2.35; 95% CI: 1.33–4.17; *p* = 0.003); total dose [hazard ratio (HR): 0.44; 95% CI: 0.27–0.71; *p* = 0.001); and pre-RT sarcopenia (HR: 2.38; 95% CI: 1.53–3.70; *p* < 0.001) were identified as independent prognostic factors for OS.

In addition, univariate and multivariate analyses were performed in 57 patients without pre-RT sarcopenia. As a result, lower total dose (HR: 0.46; 95% CI: 0.24–0.89; *p* = 0.021), higher BMI (HR: 2.39; 95% CI: 1.22–4.71; *p* = 0.011), and newly developed sarcopenia after RT (HR: 2.53; 95% CI: 1.28–5.02; *p* = 0.008) significantly affected survival in both univariate and multivariate analyses.

### Risk Factors for Newly Developed Sarcopenia After RT

We performed logistic regression analysis to investigate the risk factors associated with patients with newly developed sarcopenia after RT (Table 3). Older age [odd ratio (OR): 310.19; *p* = 0.007]; Child-Pugh classification B or C (OR: 15.24; *p* = 0.047); higher AFP (OR: 128.49; *p* = 0.008); higher PIVKA-II (OR: 118.54; *p* = 0.027); and larger PTV (OR: 51.31; *p* = 0.026) were significant factors for newly developed sarcopenia after RT.

**Table 3:** Multivariate logistic regression analysis of newly developed sarcopenia after RT.

**Risk factor**	**Regression coefficient**	**Standard error**	**Wald χ^2^ value**	***P*-value**	**Odds ratios (OR)**	**95% CI of OR**	
						**Lower**	**Upper**
Age (<65 vs. ≥65 years)	5.708	2.121	7.240	0.007	301.190	4.713	19249.211
Sex (male vs. female)	−4.467	2.388	3.498	0.061	0.011	0.000	1.238
Viral infection (No vs. Yes)	3.347	1.833	3.334	0.068	28.428	0.782	1033.409
HCC stage (I + II vs. III + IV)	−1.037	2.463	0.177	0.674	0.355	0.003	44.254
Portal vein tumor thrombus (No vs. Yes)	0.369	1.458	0.064	0.801	1.446	0.083	25.206
Child-Pugh class (A vs. B + C)	2.724	1.370	3.951	0.047	15.239	1.039	223.539
AFP (<20 vs. ≥20 ng/mL)	4.856	1.819	7.129	0.008	128.486	3.638	4537.747
PIVKA-II (<40 vs. ≥40 mAU/mL)	4.775	2.164	4.868	0.027	118.536	1.705	8242.149
ALBI score	1.489	1.282	1.349	0.245	4.433	0.359	54.710
NLR (<4 vs. 4≥)	−1.586	1.437	1.218	0.270	0.205	0.012	3.422
BMI (<25 vs. ≥25 kg/m^2^)	−1.939	1.445	1.801	0.180	0.144	0.008	2.442
Prior local treatment (No vs. Yes)	0.897	2.010	0.199	0.655	2.452	0.048	125.925
RT aim (curative vs. palliative)	−1.497	1.727	0.751	0.386	0.224	0.008	6.612
Treatment scheme (RT alone vs. CCRT)	−1.412	1.662	0.722	0.396	0.244	0.009	6.335
RT modality (3D CRT vs. IMRT + Tomo)	2.063	1.634	1.593	0.207	7.866	0.320	193.538
Total dose (EQD2 <52.1 vs. ≥52.1 Gy)	−1.564	1.593	0.964	0.326	0.209	0.009	4.748
PTV (<500 vs. ≥500 cc)	3.938	1.765	4.979	0.026	51.310	1.614	1630.973

*RT, Radiotherapy; OR, Odds ratio; CI, Confidence interval; HCC, Hepatocellular carcinoma; AFP, Alpha-fetoprotein; PIVKA-II, Proteins induced by vitamin K absence or antagonist-II; ALBI, Albumin-bilirubin; NLR, Neutrophil-lymphocyte ratio; BMI, Body mass index; CCRT, Concurrent chemoradiation therapy; 3D-CRT, 3-Dimensional conformal radiation therapy; IMRT, Intensity-modulated radiation therapy; EQD2, Equivalent dose in 2 Gy fractions; PTV, Planning target volume*.

## Discussion

We showed that sarcopenia is an independent factor affecting survival rates in HCC patients who have received RT to liver. In addition, pre-RT sarcopenia and newly developed sarcopenia after RT was significantly associated with poor survival. Patients who were older and had higher PIVKA-II level before treatment were more likely to develop sarcopenia after RT.

Recently, many hospitals have been using sarcopenia as a predictor of prognosis in cancer patients instead of cachexia, which better reflects nutrition and general condition. The European Working Group on Sarcopenia in the Elderly recommended the use of the presence of both reduced muscle mass and muscle function to identify sarcopenia (25). Methods for measuring muscle mass include measuring the total skeletal muscle mass using dual energy X-ray absorptiometry or obtaining L3-SMI through CT. Muscle function can be determined by measuring gait speed or handgrip strength. However, muscle function has the disadvantage of a difficult and subjective measurement, and several studies use only muscle mass measurement. In our study, we measured the skeletal muscle mass through CT, since this makes it easy to diagnose pre-RT sarcopenia, because the simulation CT is always taken for RT. If sarcopenia is observed on simulation CT performed for RT, we can expect that the prognosis of RT will be poor.

The hypothesis that liver disease induces sarcopenia has been reported in several studies. There are several studies showing that sarcopenia is significantly increased in patients who undergo surgery, received chemotherapy, or local therapy in HCC (18–20). Liver cirrhosis also has a similar relationship (26). Although the mechanism of this association has not been elucidated yet, low hepatic functional reserve and protein energy malnutrition due to liver disease may be the main causes of sarcopenia. In addition, reduced protein synthesis and degeneration of proteins by pro-inflammatory cytokines released from tumor cells may lead to a decrease in the amount of skeletal muscle mass. Our finding, which showed sarcopenia at a lower serum albumin and total protein level, may be attributed to protein energy malnutrition, consistent with previous studies (18–20, 26). However, Child-Pugh classification and ALBI score, which more accurately reflects liver function, did not differ between the two (Pre-RT sarcopenic and non-sarcopenic) groups (Table 1). In addition, higher PIVKA-II level, was presumed to be related to tumor burden, but the pathophysiological mechanism is also unknown. An additional biological approach would be needed to explain this result.

Chemotherapy, which is used for cancer treatment, is also suggested to induce sarcopenia by decreasing protein synthesis by expressing molecules (such as Ras, Raf, MEK, and ER) and decreasing proliferation of muscle-cell by expressing mTOR (27, 28). Like the observations in those studies, Pre-RT sarcopenia group had higher percentage of patients who underwent systemic chemotherapy before receiving RT in our study. Other studies have also shown that sarcopenia is significantly more frequent in advanced renal cell carcinoma or HCC patients with sorafenib, which is consistent with our findings (29, 30). However, in multivariate logistic regression analysis, previous systemic chemotherapy was not a significant factor, and this might have been due to the small number of patients who received chemotherapy prior to RT.

In some studies, sarcopenia has been suggested to be due to decreased appetite, general condition, and dysphagia (31, 32). In our study, sarcopenic patients have a lower BMI than non-sarcopenic patients. Because of this result, it may seem that there is no difference between sarcopenia and cachexia. However, sarcopenia and cachexia are independent because only about 4% of sarcopenic patients were underweight status (BMI <18.5 kg/m^2^).

Several studies have also been published regarding the fact that sarcopenia causes poor prognosis in the treatment of cancer. Sarcopenia is significantly associated with prognosis of cancer treatment in colon, breast, lung, head and neck cancer, and HCC (13–15). Especially in HCC, prognosis is poor when sarcopenia is present in patients receiving surgery, chemotherapy, and or local therapy (TACE, TACI, RFA) (18–20). This tendency is found not only in HCC but also in liver diseases such as liver cirrhosis, hepatitis, and liver transplantation (26, 33, 34). Our study also showed that survival was significantly poor when sarcopenia accompanied HCC. The mechanism by which sarcopenia causes poor prognosis in cancer treatment is not yet known. At present, a possible mechanism is that sarcopenia may reduce muscle strength, resulting in poor physical performance, which may reduce tolerability for cancer treatment (14). In addition, another possible mechanism is that as the amount of stored protein decreases due to sarcopenia, the metabolism and immunity decrease proportionally to this, leading to a decrease in antitumor response and an increase in mortality (35). However, the molecular mechanism of sarcopenia remains poorly understood, and further studies are needed.

In our study, univariate and multivariate analyses of OS showed that ALBI score, total irradiated dose, and pre-RT sarcopenia were significant factors. OS was significantly better when the total dose was higher than EQD2 52.1 Gy (median dose), which was in line with previous findings (36).

Consistent with the hypothesis that the low hepatic functional reserve will cause sarcopenia, ALBI score was significantly associated with survival. Compared to Child-Pugh classification, which scores clinical measures including encephalopathy, ALBI only scores objective values (albumin and bilirubin). Therefore, other studies have recently shown that the ALBI score can be used to evaluate the liver function more accurately than Child-Pugh classification (37, 38).

Among those who did not have pre-RT sarcopenia, 20 patients had newly developed sarcopenia after receiving RT. These 20 patients were significantly older, higher Child-Pugh classification B or C, higher level of AFP and PIVKA-II, and larger PTV than in patients who still had no sarcopenia after RT. Older age effect seems to be accounted for by age-related degradation of metabolism and RT tolerance. AFP and PIVKA-II may also be related to tumor burden, but the pathophysiologic mechanism is still unknown. Child-Pugh classification is a factor that is related to liver function. The larger the PTV, the greater the effect on liver function. It is very important to set up the RT field in these patients because older age, decreased liver function, and larger tumor burden eventually decrease the patient's nutritional status and reduce the muscle mass. Similarly, the total functional liver volume in sarcopenia patients is small and should be considered in surgery, when resection is performed (39). Therefore, from the radiation oncologist's point of view, if the patient needs large volume treatment, there is need to confirm whether these conditions exist in the patients.

Patients with sarcopenia prior to RT, and those newly developing sarcopenia after RT had significantly lower OS than patients who had no sarcopenia before and after receiving RT. One possibility is that sarcopenia may occur due to low hepatic functional reserve induced by progression of HCC, which may result in lower OS. Therefore, in addition to patients who have already developed sarcopenia prior to RT, patients at high risk of sarcopenia after RT—that is those of older age, with Child-Pugh classification B or C, higher AFP and PIVKA-II, larger PTV, and poor prognostic factor of HCC—also need intensive nutritional support during RT to prevent the development of sarcopenia after treatment. According to the study conducted in our hospital, repetitive and intensive nutritional counseling is needed to improve the quality of life and prevent deterioration of nutritional status in patients with cancer around the head and neck, thorax, and abdomen receiving RT (40). Nutritional interventions in HCC are also expected to show good results in sarcopenia patients. In our prospective study on the effect of oral supplementation in HCC patients undergoing RT, we found that serum albumin levels increased significantly in the group given oral supplementation with branched-chain amino acid (41). Therefore, oral supplementation in patients receiving RT for HCC is very important, in terms of nutritional support. In our hospital, nutritional counseling is given to all patients regardless of sarcopenia before RT in HCC patients. This may likely improve survival rate in these patients.

One of the limitations of this study was that because of the retrospective analysis, not all variables and possible confounders could be assessed in all patients. In our study, pre-RT sarcopenic patients group was significantly larger in PTV than the non-sarcopenic group. Therefore, this result is probably related to the higher RT dose in non-sarcopenia patients. In Cox regression analysis to determine the OS-related prognostic factors (Table 2), RT dose had a significant effect on OS, but not PTV volume. In patients with low disease extent and good performance status, the treatment volume would be small and such patients could be given a high dose. Likewise, patients' characteristics and treatment were heterogeneous, suggesting the possibility that various confounding factors may exist, which is considered a limitation of retrospective study.

There were also significant differences in previous chemotherapy, RT dose, serum albumin level, and total protein level between patients with and without pre-RT sarcopenia. This is because the degree of disease had varied considerably by the time the HCC patients reached the point of considering RT. However, there were no significant differences in indicators of liver function such as Child-Pugh classification and ALBI score. Moreover, there were no clinically significant differences between each of the two groups in Table 1 (Pre-RT sarcopenic and non-sarcopenic) and Supplementary Table 1 (Newly developed sarcopenic and sustained non-sarcopenic) characteristics. Nevertheless, these findings could be due to selection bias and unmeasured confounders and are considered to be the limitations of this study. Therefore, from a physician's point of view, efforts such as matching based on these factors may help to reduce the selection bias and unmeasured confounders. However, the disease status and treatment were too heterogeneous relative to the number of patients, making matching difficult. In addition, we did not analyze the treatment after RT, and further analysis is needed because it may also affect prognosis. Nonetheless, this study is one of the largest analyses to assess the prognostic significance of sarcopenia in patients with HCC who received RT. Also, as far as we know, the strength of this study is that it is one of the few studies that analyzed the prognostic significance of sarcopenia in cancer patients receiving RT, especially in patients with HCC.

## Conclusion

In conclusion, the findings of this study suggest that sarcopenia is frequent in patients with HCC, especially in those with lower liver function, higher tumor markers, and larger PTV; thus, these patients are more likely to develop sarcopenia after RT. Thus, the RT field should be carefully determined for these patients from the point of view of the radiation oncologist. Assessment of skeletal muscle depletion by CT imaging is an objective tool useful for diagnosing sarcopenia. In addition, pre-RT sarcopenia and newly developed sarcopenia after RT, as determined by simulation CT for RT, was significantly associated with poorer OS in HCC patients. Early intervention such as nutritional support and exercise therapies could prevent muscle wasting and could be effective in improving the prognosis of HCC patients. Prospective studies are needed to clarify optimum reference values of sarcopenia for predicting cancer-specific outcomes for HCC patients.

## Data Availability Statement

The datasets generated for this study are available on request to the corresponding author.

## Ethics Statement

The studies involving human participants were reviewed and approved by The Institutional Review Board of the Yonsei University Health System (No. 3-2019-0039). Written informed consent for participation was not required for this study in accordance with the national legislation and the institutional requirements.

## Author Contributions

JL, YC, and IL: study concept and design and manuscript preparation. JK and IL: data acquisition and quality control of data. JL, YC, SP, and IL: data analysis and interpretation. SP and JK: manuscript review.

### Conflict of Interest

The authors declare that the research was conducted in the absence of any commercial or financial relationships that could be construed as a potential conflict of interest.
