# Supplementary material for: Skeletal Muscle Depletion Predicts the Prognosis of Patients With Hepatocellular Carcinoma Treated With Radiotherapy
Source: Front Oncol. 2019 Oct 15;9:1075. doi: 10.3389/fonc.2019.01075 (PMC6803501; doi:10.3389/fonc.2019.01075)
Supplement: Supplementary file 1 [file Table_1.docx]

Supplementary Material

Supplementary table 1. Characteristics of patients without pre-RT sarcopenia according to the presence of post-RT sarcopenia.

|  |  | Pre-RT non-sarcopenic | | | | |
| --- | --- | --- | --- | --- | --- | --- |
|  |  | Post-RT non-sarcopenic | | Post-RT sarcopenic | | p-value |
|  |  | N = 35 | *%* | N = 20 | *%* |  |
| Age | Median | 53 | | 61 | | 0.027 |
|  | Range | 34-80 | | 40-80 | |  |
| Sex | Male | 28 | 80.0 | 17 | 85.0 | 0.731 |
|  | Female | 7 | 20.0 | 3 | 15.0 |  |
| Etiology | HBV | 24 | 68.6 | 14 | 70.0 | 0.949 |
|  | HCV | 5 | 14.3 | 2 | 10.0 |  |
|  | Non-B, non-C | 6 | 17.1 | 4 | 20.0 |  |
| HCC stage | I | 2 | 5.7 | 0 | 0.0 | 0.395 |
|  | II | 5 | 14.3 | 1 | 5.0 |  |
|  | III | 10 | 28.6 | 8 | 40.0 |  |
|  | IVA | 14 | 40.0 | 9 | 45.0 |  |
|  | IVB | 4 | 11.4 | 2 | 10.0 |  |
| Portal vein tumor thrombus | Yes | 18 | 51.4 | 15 | 75.0 | 0.086 |
|  | No | 17 | 48.6 | 5 | 25.0 |  |
| Child-Pugh score | A | 26 | 74.3 | 12 | 60.0 | 0.178 |
|  | B | 9 | 25.7 | 7 | 35.0 |  |
|  | C | 0 | 0.0 | 1 | 5.0 |  |
| AFP (ng/mL) | Median | 19.9 | | 383.7 | | 0.027 |
|  | Range | 1.8-54000 | | 2.0-54000 | |  |
| PIVKA-II (mAU/mL) | Median | 347.0 | | 587.0 | | 0.087 |
|  | Range | 17.0-37814 | | 17.0-19347 | |  |
| Total protein (g/dL) | Median | 7.0 | | 6.9 | | 0.616 |
|  | Range | 5.4-8.5 | | 5.6-8.4 | |  |
| Albumin (g/dL) | Median | 3.6 | | 3.6 | | 0.148 |
|  | Range | 2.5-4.8 | | 2.9-4.1 | |  |
| ALBI score | Median | -2.35 | | -2.22 | | 0.098 |
|  | Range | -3.37 to -0.91 | | -2.59 to -1.60 | |  |
| BMI (kg/m²) | Underweight | 0 | 0.0 | 0 | 0.0 | 0.474 |
|  | Normal weight | 15 | 42.9 | 11 | 55.0 |  |
|  | Overweight | 16 | 45.7 | 7 | 35.0 |  |
|  | Obesity | 4 | 11.4 | 2 | 10.0 |  |
| Previous treatment | Surgery | 4 | 11.4 | 3 | 15.0 | 0.702 |
|  | Chemotherapy | 1 | 2.9 | 0 | 0.0 | 0.446 |
|  | TACE/TACI/RFA | 24 | 68.6 | 13 | 65.0 | 0.786 |
|  | None | 9 | 25.7 | 7 | 35.0 | 0.466 |
| RT aim | Curative | 29 | 82.9 | 14 | 70.0 | 0.319 |
|  | Palliative | 6 | 17.1 | 6 | 30.0 |  |
| Treatment scheme | RT alone | 22 | 62.9 | 9 | 45.0 | 0.199 |
|  | CCRT | 13 | 37.1 | 11 | 55.0 |  |
| RT modality | 3D-CRT | 13 | 37.1 | 8 | 40.0 | 0.834 |
|  | IMRT | 22 | 62.9 | 12 | 60.0 |  |
| RT dose | Median | 54.0 | | 52.1 | | 0.118 |
| (EQD2, α/β = 10) | Range | 40.7-125.0 | | 40.0-65.1 | |  |
| PTV (cc) | Median | 577.5 | | 738.6 | | 0.549 |
|  | Range | 15.0-2840.4 | | 40.8-1787.7 | |  |
| RT scheme | Conventional RT | 33 | 94.3 | 20 | 100.0 | 0.529 |
|  | SBRT | 2 | 5.7 | 0 | 0.0 |  |

RT, Radiotherapy; HBV, Hepatitis B virus; HCV, Hepatitis C virus; HCC, Hepatocellular carcinoma; AFP, Alpha-fetoprotein; PIVKA-II, Proteins induced by vitamin K absence or antagonist-II; ALBI, Albumin-bilirubin; BMI, Body mass index; TACE, Transcatheter arterial chemoembolization; TACI, Transcatheter arterial chemotherapy infusion; RFA, Radiofrequency ablation; CCRT, Concurrent chemoradiation therapy; 3D-CRT, 3-Dimensional conformal radiation therapy; IMRT, Intensity-modulated radiation therapy; EQD2, Equivalent dose in 2 Gy fractions; PTV, Planning target volume; SBRT, Stereotactic body radiotherapy
